# Microchannel Liquid-Cooled Heat Exchanger Based on a Nonuniform Lattice: Study on Structure Calculation, Formation Process, and Boiling Heat Transfer Performance

**DOI:** 10.3390/ma14237248

**Published:** 2021-11-27

**Authors:** Bo Qian, Hongri Fan, Gang Liu, Jianrui Zhang, Pei Li

**Affiliations:** 1 School of Mechanical and Automotive Engineering, Shanghai University of Engineering Science, Shanghai 201620, China; qianbo@ecust.edu.cn (B.Q.); hrfan@sues.edu.cn (H.F.); 2 School of Mechanical and Power Engineering, East China University of Science and Technology, Shanghai 200237, China; lipei_ecust@163.com

**Keywords:** nonuniform lattice, selective laser melting (SLM), microchannel heat exchanger, boiling heat transfer, cell structure

## Abstract

A microchannel radiator is advantageous due to its high efficiency and large boiling heat transfer coefficient of two-phase flow. Based on the research of uniform lattice structures, this study proposed a microchannel heat exchanger with a nonuniform lattice structure. The calculation, optimal formation, and boiling heat transfer performance of the nonuniform lattice structure based on selective laser melting (SLM) were investigated, and heat exchange samples were successfully prepared using SLM. The porosity and pore morphology of the samples were analysed, and the contrast experiments of boiling heat transfer were conducted with deionised water. The results revealed that the heat flow density of the lattice structure was a minimum of 244% higher than that of the traditional liquid-cooled plate. The critical heat flux density of the lattice structure is 110 W∙cm^−2^, and the critical heat flux density of the traditional flat plate is 45 W∙cm^−2^. In addition, the effects of cell structures indicated that for frame cells, the heat transfer effect of nonuniform frames was inferior to that of uniform frames; for face-centred cubic (FCC) cells, the nonuniform and uniform frames exhibited the same trend. However, the heat flow density of FCC cells was 25% higher than that of frame structures.

## 1. Background

Microchannel heat exchangers have a simple structure, typically with a rectangular, triangular, circular, or trapezoidal cross-section. They exhibit a small volume and thus can be directly used as a millimetre- or micron-sized heat source; they also provide high heat-exchange efficiency, and their fluid state is primarily laminar flow. Generally, a water column pressure drop of tens of millimetres of heads is sufficient. Microchannel heat exchangers can work in harsh environments and their heat transfer efficiency depends on their microchannel structure [1]. With the increase in power density, microchannel heat exchangers/radiators have made considerable progress in the past two decades; however, small devices face many heat dissipation problems [2]. Gas/liquid two-phase boiling heat transfer can eliminate more heat, is more energy-efficient, cools more uniformly, and requires lower pump power. This indicates it will be the main research direction of heat dissipation in the future, rather than single-phase convection (the liquid remains in the liquid state throughout cooling) [3] as traditional single-phase liquid cooling is not ideal under high heat fluxes. Two-phase liquid-cooled boiling heat exchangers have high efficiency and are becoming increasingly crucial in the study of high-power equipment cooling [4,5].

With its high machining flexibility, selective laser melting (SLM), which can realise the efficient formation of complex microchannel heat exchangers, has been used to produce heat exchangers [6]. SLM is an advanced manufacturing technology developed in the 1990s [7,8]. In SLM, according to the principles of additive manufacturing, a computer-aided design model is sliced and layered, and a laser scans the paths determined using layering software. By melting metal powders with the high-power laser and stacking them layer by layer, rapid, completely dense, and near-net formation can be achieved for high-performance metal parts with complex structures [9]. SLM can produce parts with higher surface roughness than traditional manufacturing methods, which can increase the surface area in the same size. These features enable highly efficient heat exchange in SLM-manufactured heat exchangers. Conventional heat exchangers expand the surface area with pins and fins [10]. The effect of surface area expansion can be considerably enhanced using SLM [11,12]. In addition, geometric features on the parts can strengthen flow mixing, thereby further improving heat dissipation [13]. Another advantage of SLM is composite structure integration or multi-material manufacturing [14], which can optimise characteristics such as thermal conductivity and allow integration with other heat dissipation components. Therefore, the use of SLM in functional heat transfer components, such as heat exchangers, heat sinks, and heat pipes, has attracted increased attention [15,16].

With a low density and remarkable mechanical properties, lattice structures are applied in aerospace, medicine, biology, and chemical engineering fields [15]. A three-dimensional (3D) porous lattice, with a structure similar to the structure of the space lattice, was first proposed by Evans et al. [16] of Harvard University in 2000. Due to their high specific strength and stiffness, porous structures can be used in many environments, especially when lightweight designs are needed [17]. Nowadays, the use of additive technologies, such as SLM, to manufacture lattice structures has become a research focus [18]. SLM enables the accurate manufacture of lattice structures and control of the relative density, pore size, opening position, and connection of each pore. SLM ensures the mechanical properties of parts while reducing the weight and achieving excellent heat transfer. The SLM-manufactured lattice structure is competitive if light-weight and high-modulus-ratio mechanical bearing parts are desired. In this study, according to the special process requirements of additive manufacturing, a supporting rod in the lattice structure was considered a spatial frame. Many scholars have analysed the performance of the supporting rod mechanical structure. On the basis of fundamental mechanical properties of lattice structures, Brooks was the first scholar to systematically study the mechanical properties of the SLM-manufactured lattice structure in 2005 [19]. Deshpande et al. [20] studied the performance of a lattice structure dominated by tensile loads, Wang et al. [21] investigated the performance of a lattice plate based on the 3D Kagome structure, and Son Pham et al. [22] explored the influence of the internal cell arrangement on the mechanical properties of porous structures.

A lattice is a porous structure formed through the periodic combination of numerous cells of the same component. The lattice provides order and periodicity on the mesoscopic scale and a high surface volume ratio for heat transfer. The lattice structure exhibits a unique advantage in heat exchangers, and SLM-manufactured lattices are used for microchannel liquid-cooled heat transfer structures to withstand high heat fluxes [23]. A two-phase wick constituting lattice structures is the key to microchannel liquid-cooled heat exchangers. Therefore, improving the boiling heat transfer characteristics of microchannel lattice structures is important for developing new liquid coolers. The lattice structure must provide a sufficient capillary force to overcome the liquid flow resistance. Its material properties (e.g., corrosion resistance and thermal conductivity), size design, and structural parameters (e.g., porosity, average pore diameter, and pore distribution) can considerably affect the heat transfer performance of liquid coolers [24,25]. However, studies on the SLM-formed lattice structure have evaluated mechanical properties with limited focus on the boiling heat transfer characteristics of these structures. Moreover, matching between the size parameters of the lattice structure and the heat flow path of the heat exchange fluid is not discussed in detail. In the design of nonuniform lattices, the lightweight idea and topology optimisation theory are combined. The current design and manufacturing of lightweight lattices mostly employ uniform units, which exhibit the same internal structure parameters and arrangement in a single component, with an even density. This nonuniform lattice design, however, is unhelpful under uneven external heat conduction and nonuniform heat flow. By designing nonuniform lattices according to the optimised theoretical results [26,27], the adaptability of lattice structures to the requirements of two-phase boiling heat transfer can be improved while enhancing heat transfer and dissipation efficiencies. This is the first study to systematically investigate the applications of SLM-formed nonuniform lattices in boiling heat transfer and explore structure calculations and formation through the combination of SLM formation with the requirements of the two-phase microchannel liquid-cooled heat transfer structure. The effect of structure on bubble escape and liquid replenishment in boiling heat transfer was analysed, which explained why boiling heat transfer enhancement differed with varying nonuniform lattice structures. The optimal structures were obtained under different heat flow densities, providing a comprehensive reference for manufacturing high-density two-phase heat transfer structures. Additionally, the nonuniform lattice Liquid-Cooled heat exchanger in this paper can be potentially applied to the field of surface heat dissipation of heat exchangers, radiators, and small electronic devices.

## 2. Calculation

To construct the proposed nonuniform lattice heat exchanger, an algorithm was developed according to the characteristics of the two-phase heat exchanger to obtain nonuniform lattices of any size. First, the lattice parameters were configured according to the overall size of the heat exchanger. Next, the cell structure inside the lattice was calculated according to the acquired lattice parameters. Subsequently, for additive manufacturing, the slice contour data were computed based on the calculated cell structure. The specific calculation process is as follows.

### 2.1. Data Structure and Function Definitions

(1) M—3D part model input in a standard template library (STL) format.
(2) $G_{L}{}_{1}$, $G_{L}{}_{2}$, $G_{h}$.

**Figure 1:**
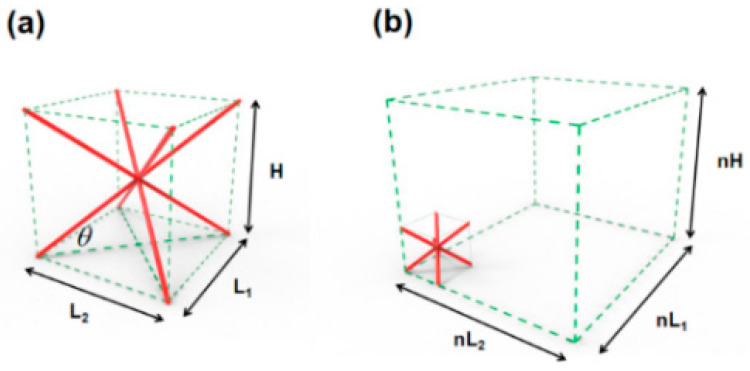
BCC lattice structure. (**a**) Composition of the body-centred cubic (BCC)-type single-cell supporting rod and (**b**) Spatial arrangement of a single cell in the lattice.

**Definition** **1.** *The cell spacing values (i.e., cell length, width, and height) in the lattice structure, as shown in Figure 1.*

(3) The equation for calculating the space range of the bounding box in the model. Specifically, for the three directions, calculate the respective maximum values, $X_{\max}$, $Y_{\max}$, and $Z_{\max}$; minimum values, $X_{\min}$, $Y_{\min}$, and $Z_{\min}$; and lengths, $L_{x}$, $L_{y}$, and $L_{z}$;

(1) C = Cube(M)

(4) The equation for calculating the number of cells in X and Y directions according to the range of the bounding box.

(2) $$X_{count}\;=\;\left[L_{x}/G_{L}{}_{1}\right]\;+\;1,\;Y_{count}\;=\;\left[L_{y}/G_{L}{}_{2}\right]\;+\;1$$

(5) $\left(X_{cent},\;Y_{cent},\;Z_{cent}\right)$.

**Definition** **2.** *Coordinates for the centre point in the cell structure.*

(6) The equation for calculating the four corners of the lower cell frame according to the coordinates of the centre point.

(3) $$\left\{\begin{array}{l} P_{000}:(X_{cent}\;-\;G_{L}{}_{1}/2,Y_{cent}\;-\;G_{L}{}_{2}/2,Z_{cent}\;-\;G_{h}/2)\\ P_{010}:(X_{cent}\;-\;G_{L}{}_{1}/2,Y_{cent}+G_{L}{}_{2}/2,Z_{cent}\;-\;G_{h}/2)\\ P_{100}:(X_{cent}\;+\;G_{L}{}_{1}/2,Y_{cent}\;-\;G_{L}{}_{2}/2,Z_{cent}\;-\;G_{h}/2)\\ P_{110}:(X_{cent}\;+\;G_{L}{}_{1}/2,Y_{cent}\;+\;G_{L}{}_{2}/2,Z_{cent}\;-\;G_{h}/2) \end{array}\right\}$$

(7) The equation for calculating the four corners of the upper cell frame according to the coordinates of the centre point.

(4) $$\left\{\begin{array}{l} P_{001}:(X_{cent}\;-\;G_{L}{}_{1}/2,Y_{cent}\;-\;G_{L}{}_{2}/2,Z_{cent}\;+\;G_{h}/2)\\ P_{011}:(X_{cent}\;-\;G_{L}{}_{1}/2,Y_{cent}\;+\;G_{L}{}_{2}/2,Z_{cent}\;+\;G_{h}/2)\\ P_{101}:(X_{cent}\;+\;G_{L}{}_{1}/2,Y_{cent}\;-\;G_{L}{}_{2}/2,Z_{cent}\;+\;G_{h}/2)\\ P_{111}:(X_{cent}\;+\;G_{L}{}_{1}/2,Y_{cent}\;+\;G_{L}{}_{2}/2,Z_{cent}\;+\;G_{h}/2) \end{array}\right\}$$

(8) Δh.

**Definition** **3.** *Cell height variation in a nonuniform lattice.*

(9) Indicates the level of non-uniformity for the nonuniform lattice structures.

(5) $$Lv_{UL}{}_{Z}\;=\;\left|\frac{\Delta h}{G_{h}}\right|\;\times \;100\%$$

### 2.2. Calculation Method of the Nonuniform Lattice Structure

Step 1: Calculate the overall length, width, and height, that is, $L_{x}$, $L_{y}$, and $L_{z}$, of the lattice structure with the known model according to Equation (1), and set the length, width, and height of the unit cell.

Step 2: Calculate the number of cells in the X and Y directions by using Equation (2) according to the overall lattice and single-cell lengths and widths. Define current height $H_{state}$, and initialize it as $H_{state}=0$.

Step 3: Determine whether the current height is less than $Z_{\max}$. If yes, enter the next iteration. Otherwise, exit the iteration.

**Figure 2:**
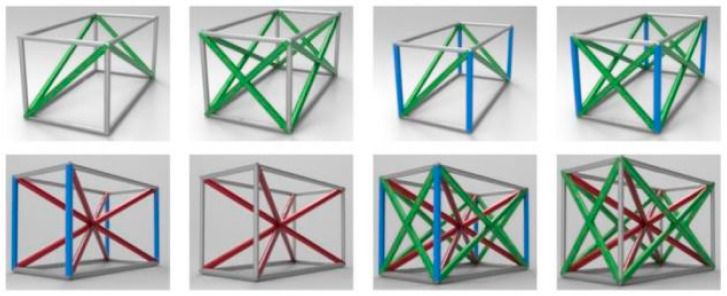
Cell structure types of face-centred cubic (FCC), BCC, and FCC/BCC hybrid structures used in a nonuniform lattice.

Step 4: Calculate the cell arrays in the X and Y directions at the current height $H_{state}$ according to the set lattice structure type. The structure types include the face-centred cubic (FCC) cell, body-centred cubic (BCC) cell, and FCC/BCC hybrid [19] (Figure 2). Specifically, for FCC, the procedure is as follows:

(1) Calculate the X- and Y-direction cell arrays one by one. Define the current X-direction sequence number as $X_{i}$ and Y-direction sequence number as $Y_{j}$; both of their initial values are 0.

(2) Use the two-layer traversal method to increase the values of $X_{i}$ and $Y_{j}$ in sequence. The centre point of an X-row and Y-column cell structure is calculated as follows: $X_{cent}{\;=\;X}_{\min}\;+\;X_{i}\cdot G_{L}{}_{1}$, $Y_{cent}\;=\;Y_{\min}\;+\;Y_{j}\cdot G_{L}{}_{2}$, and $Z_{cent}\;=\;Z_{\min}\;+\;H_{state}$.

(3) Calculate the four corners of the lower and upper cell frames according to Equations (3) and (4).
(4) Calculate the supporting rod segments of the cell according to the cell structure type. If it has an FCC structure, the supporting rod segments include eight horizontal frame lines, eight inclined cross lines in vertical planes, four vertical frame lines, and four inclined cross lines in horizontal planes. For a BCC structure, the supporting rod segments are eight horizontal frame lines, four vertical frame lines, and four inclined cross lines inside the cube.

Step 5: Increase current height $H_{state}$. If the gradient increased, $H_{newstate}\;=\;H_{state}\;+\;G_{h}\;+\;\Delta h$; if the gradient decreased, $H_{newstate}\;=\;H_{state}\;+\;G_{h}\;-\;\Delta h$. Assign new height $H_{newstate}$ to current height $H_{state}$($H_{state}\;=\;H_{newstate}$), and return to Step 3 for iteration.

Step 6: Perform cutting for all supporting rod segments. Specifically, if a part of the segment crosses the bound, cut that part, and store the remaining part in the cell structure. If all the segments are beyond the model space, delete it. If the segment is completely inside the model space, skip to the next segment. Then generate a triangular patch entity of each supporting rod according to its diameter. If the diameter of the supporting rod continuously changes, increase or decrease the rod diameter accordingly when outputting the STL model.

**Figure 3:**
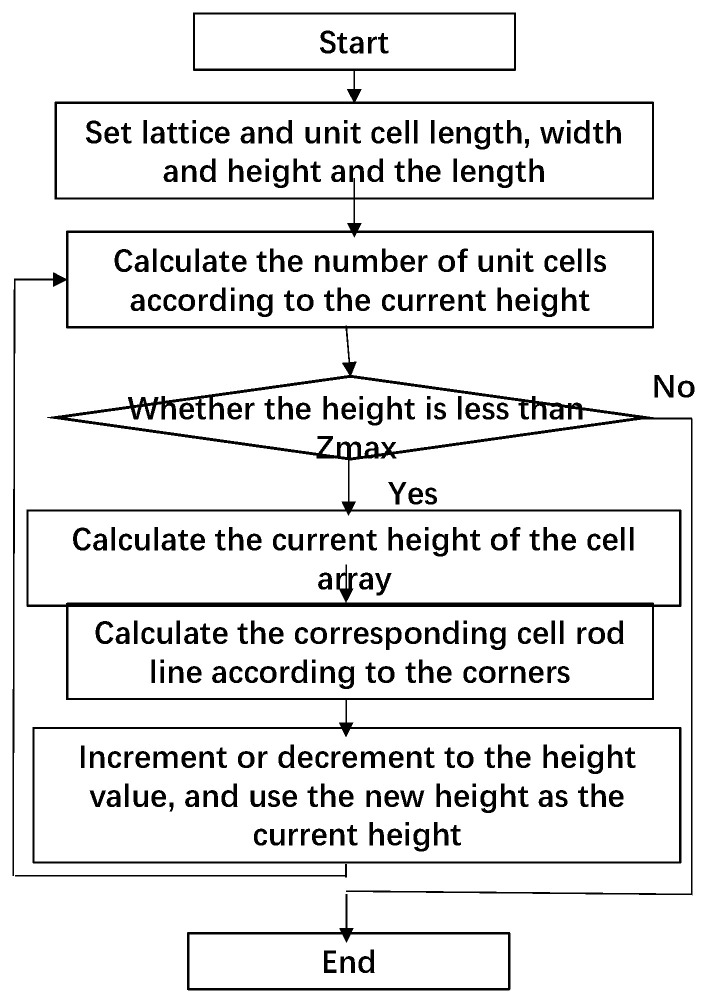
Calculation steps for the nonuniform lattice structure.

All the cell structures and cell supporting rod segments in the model space can be calculated using the aforementioned six steps, as indicated by the following flow chart, as shown in Figure 3:

### 2.3. Calculation Examples

**Figure 4:**
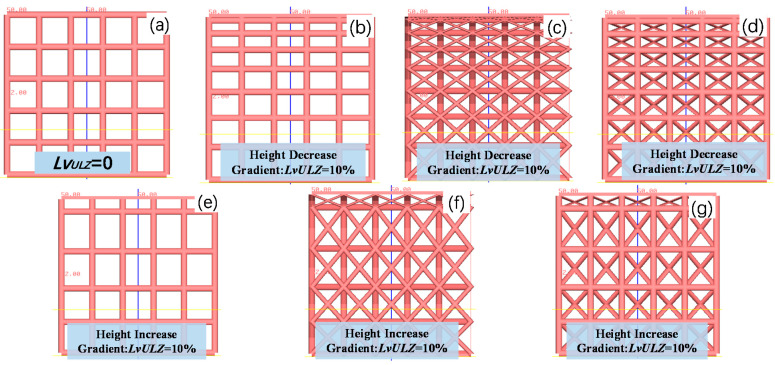
Calculation results for six nonuniform lattice structures and one uniform lattice structure. (**a**) Uniform frame structure, (**b**) Frame structure with a height decreasing gradient of 1 mm and height decreasing gradient 10%, (**c**) Face CC type with a height decreasing gradient of 1 mm and height decreasing gradient 10%, (**d**) BCC hybrid type with a height decreasing gradient of 1 mm and height decreasing gradient 10%, (**e**) Frame structure with a height increasing gradient of 1 mm and height increasing gradient 10%, (**f**) FCC type with a height increasing gradient of 1 mm and height increasing gradient 10%, and (**g**) BCC hybrid type with a height increasing gradient of 1 mm and height increasing gradient 10%.

The calculation method was verified using the following parameters. The lattice size was set to 50 mm × 50 mm × 50 mm, the cell dimension was 10 mm × 10 mm × 10 mm, and the diameter of the supporting rod was 0.5 mm. The cell height was decreased or increased by 1 mm at step one, and the cell types were set as the frame structure, FCC type, and BCC type. The calculated nonuniform lattice models and the non-uniformity are presented in Figure 4. Additionally, the height decreasing or increasing gradient in the figure is the non-uniformity in the Z direction, and the calculation formula refers to Equation (5).

## 3. Experimental Methods and Materials

### 3.1. Experimental Platform

**Figure 5:**
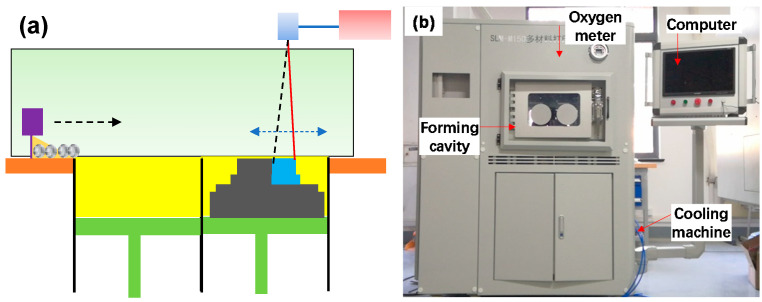
Schematic of the SLM equipment and experimental facility [28]. (**a**) Schematic diagram of the SLM forming and (**b**) Actual SLM equipment.

The schematic diagram of the host is shown in Figure 5a. The SLM experimental facility constituted a host, laser system, cooling system, control system, and gas protection system (Figure 5b) [28]. The laser was an RFL-C300L continuous-wave fibre laser. The laser parameters are presented in Table 1. The printer had a formation size of 150 mm × 150 mm × 120 mm and fed powders through a cylinder.

### 3.2. Experimental Platform of Boiling Heat Transfer and Its Calculation Method

**Figure 6:**
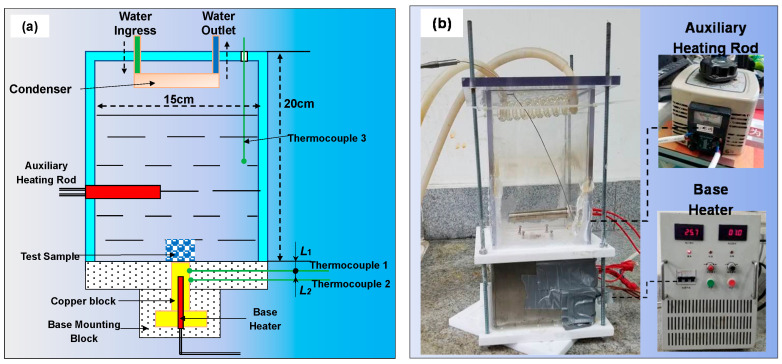
Experimental setup for boiling heat transfer. (**a**) Schematic diagram of the experimental platform and (**b**) Actual experimental platform.

The experimental boiling heat transfer platform included a boiling heat transfer test device, an auxiliary heating control device, the main heating control device, and a data acquisition device (Figure 6) [28]. An auxiliary heating rod and a condensing tube were installed for preheating and condensing, respectively. These steps were followed in the boiling heat transfer experiment: The boiling working medium was added into the container, the power of the auxiliary heating rod was adjusted, and the liquid was heated in the container to the saturated boiling point for approximately 30 min to eliminate noncondensable gases and the gases present in sample pores [28]. The main heater was turned on to a specific power, and the temperature data using the thermocouples were recorded when the system reached the quasi-steady state. The heating power of the main heating rod was gradually increased; whether the data of the thermocouples attained the stable state was monitored, and the collected data were recorded [28].

The heat flux *q*/W∙cm^−2^ is calculated as follows:

(6) $$q\;=\;\lambda_{\mathrm{Cu}}\frac{T_{2}\;-\;T_{1}}{L_{2}}\;\times \;10^{4}$$

The wall temperature *T*_w_/°C of the copper block is calculated as follows [29]:

(7) $$T_{W}\;=\;T_{1}\;-\;\frac{T_{2}\;-\;T_{1}}{L_{2}}\;\times \;L_{1}$$

### 3.3. Experimental Materials

The experimental material used was a copper alloy, CuSn10. Its chemical composition is presented in Table 2. Its particle size distribution was as follows: 96% sphericity, 398 ppm oxygen content, particle size D10 (23.56 μm), particle size D50 (31.62 μm), and particle size D90 (52.83 μm).

## 4. Preparation, SLM Formation, and Roughness Analysis of Nonuniform Lattice Structures

### 4.1. Design and Preparation of Nonuniform Lattice Structures

**Figure 7:**
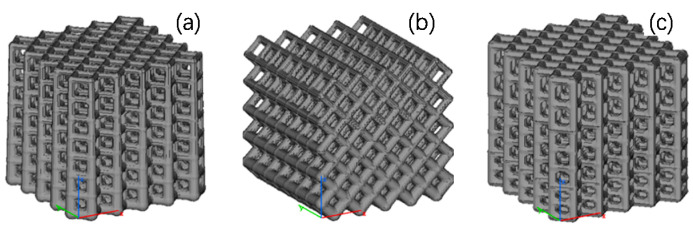

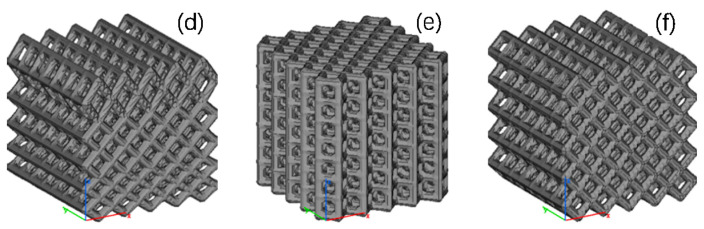
Lattice structure models. (**a**) Frame-type lattice structure C1-1 with a continuous diameter change, (**b**) Lattice structure C2-1 with a continuous diameter change, (**c**) Lattice structure C1-2 with step-change cell heights, (**d**) Lattice structure C2-2 with step-change cell heights, (**e**) Uniform lattice structure C1-3, and (**f**) Uniform lattice structure C2-3.

**Figure 8:**
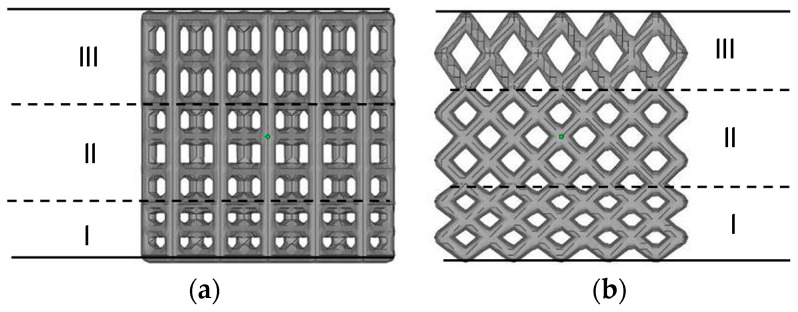
Cell structure in the height direction of nonuniform lattice structures. (**a**) C1-2 with gradient increasing cell heights and (**b**) C2-2 with gradient increasing cell heights.

According to the calculation method presented in Section 1, two nonuniform lattice heat transfer structures were prepared: the frame and FCC lattice structures with continuous rod diameter changes (Figure 7a,b) and nonuniform frame and FCC lattice structures with gradient increasing heights (Figure 7c,d). Figure 7e,f shows the uniform lattice structure with the same porosity. The nonuniform lattice structure with gradient increasing heights was divided into three parts. Parts I, II, and III of frame-type C1-2 lattice structure constituted cells with a height of 0.75 *h*, *h*, and 1.25 *h*, respectively (Figure 8a). Parts I, II, and III of FCC-type C2-2 lattice structure constituted cells with a height of 0.75 *h*, *h*, and 1.5 *h*, respectively (Figure 8b); *h* is the height of the unit cell, and its value is 10 mm.

The lattice structures generated using the aforementioned algorithm were output as STL models, and their porosities were analysed. The overall size was 15 mm × 15 mm × 15 mm, the porosities exhibited nearly the same size, and their surface areas slightly differed. The specific parameters are presented in Table 3.

**Figure 9:**
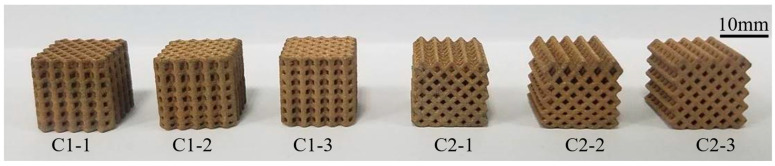
Nonuniform lattice structure samples were formed using SLM.

As a tin brass, CuSn10 has a thermal conductivity similar to the thermal conductivity of brass (118 W∙(m∙°C)^−1^), which is conducive to enhancing boiling heat transfer. However, the high thermal conductivity and low laser absorptivity of copper alloys can lead to considerable heat loss and insufficient powder melting, which causes difficulties in SLM. Specifically, the high thermal conductivity and low laser absorptivity (0.59) make the accumulation of the CuSn10 powder difficult to heat during SLM, which results in poor formation performance. The lattice structure designed in this study had a large cell size (2 mm × 2 mm × 2 mm), its rough surface was conducive to the formation of potential nucleation sites, and the required formation accuracy was low. Therefore, although the CuSn10 powder has high thermal conductivity and low laser absorptivity, it was used as the experimental material. SLM parameters are presented in Table 4, and the six formed copper-alloy nonuniform lattice liquid-cooled heat exchange parts are shown in Figure 9.

### 4.2. Analysis of the Surface Morphology and Porosity

**Figure 10:**
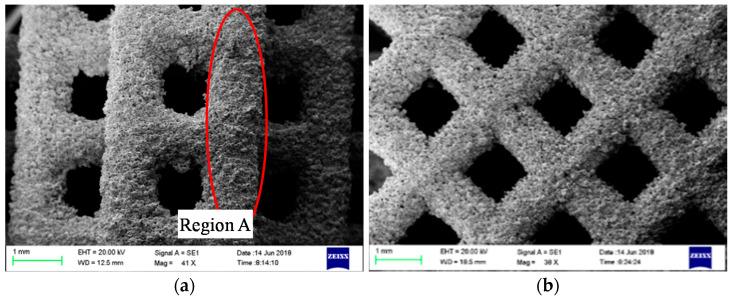
SEM images of the lattice structure samples. (**a**) C1-3 and (**b**) C2-3.

**Figure 11:**
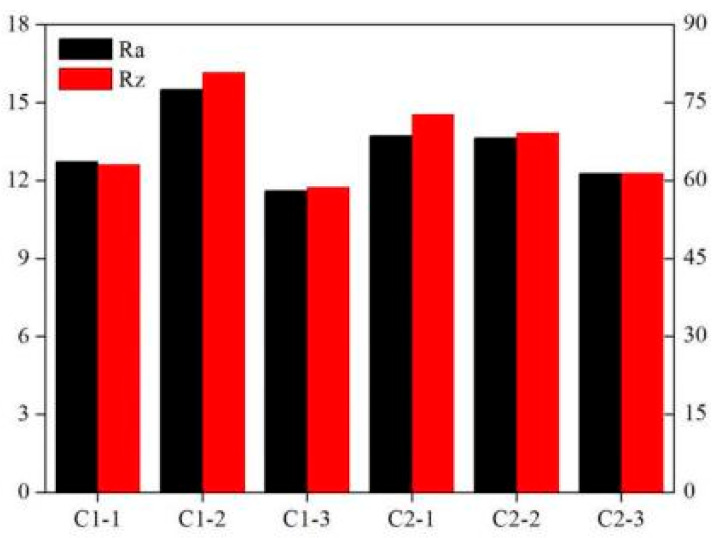
Surface roughness measurements of various samples.

The primary factors affecting the two-phase boiling heat transfer of nonuniform lattice structures include the surface area and surface morphology, which are used to determine the number of potential nucleation sites, and the structural parameters, including porosity; cell configuration; and structural changes, which affect liquid replenishment and bubble escape [30]. This study discussed the surface morphology, porosity, cell configuration, and heterogeneity of the lattice structure and their effects on two-phase boiling heat transfer. The side surface of the sample was observed through scanning electron microscopy (SEM). The results are shown in Figure 10. Large quantities of powder particles were bonded on the column surface, and slags were present on the lower surface of the horizontal column. Powder bonding led to rough sample surfaces, which facilitated the formation of potential nucleation sites. The side surface roughness was measured for the six groups of samples. The results are shown in Figure 11. The arithmetic mean deviation (Ra) and Rz values of all the sample surfaces were in the range of 10–16 μm and 55–85 μm, respectively.

**Figure 12:**
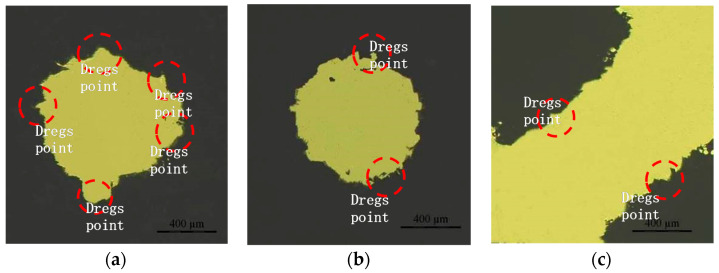
Cross-sections of columns with different angles. (**a**) Horizontal column (radial section of C2-3), (**b**) Vertical column (radial section of C1-3), and (**c**) 45° inclined column (shaft section of C2-3).

The porosity was measured by drying and weighing the samples. The results are presented in Table 5. For each sample, the actual porosity was smaller than the designed porosity mainly due to slagging, powder bonding, and the step effect during SLM. The C1-3 and C2-3 samples were polished and analysed with a microscope. During the formation of the horizontal columns, as the laser with a poor heat exchange capacity scanned the powder, the energy accumulated and large overhangs formed [31], resulting in a poor formation accuracy (Figure 12). The vertical columns exhibited some powder bonding, but their shapes were regular, and the formation accuracy was high (Figure 12b). Because of a small inclination angle (45°) and small layer thickness (50 μm), the inclined columns experienced an unobvious step effect during formation, and the formation accuracy was high (Figure 12c). Table 5 reveals that the relative porosity error for the C1-frame lattice structure was larger than that for the C2-FCC cell structure because slags, which had a greater impact on porosity than the step effect and powder bonding, were more frequent in the C1-frame sample with more horizontal columns.

## 5. Analysis of Boiling Heat Transfer Characteristics

### 5.1. Comparison of Heat Transfer Characteristics between the Nonuniform Lattice Structure and Plate

**Figure 13:**
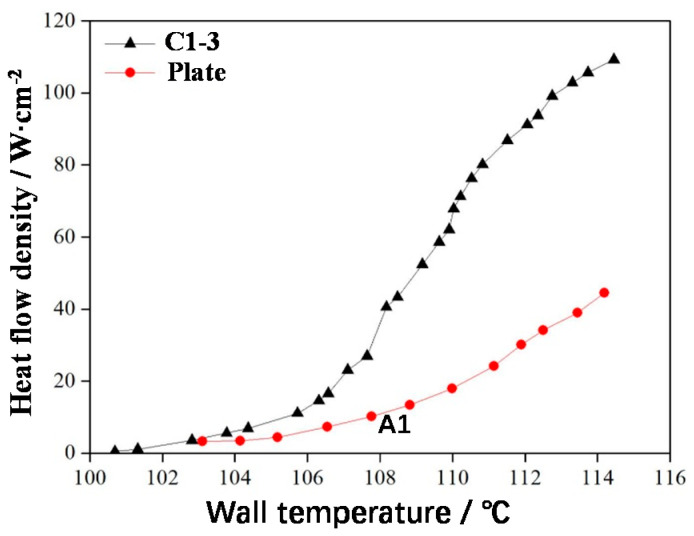
Saturated boiling curves of the lattice structure and surface samples.

According to the test method described in Section 2.2, the boiling heat transfer experiment was conducted with deionised water. The saturated boiling curves of the C1-3 sample and a smooth plate are presented in Figure 13. The boiling heat transfer process of C1-3 can be divided into the following two stages.

(1) Single-phase convection stage (starting zone)

In the starting zone, the wall temperature of the uniform frame lattice sample (C1-3) was higher than the saturation temperature of the water. However, no obvious boiling occurred because of the superheating of the small wall, i.e., a few bubbles were generated on the sample but did not float up. At this stage, the boiling curves of C1-3 and the smooth plate were similar because heat transfer at this moment depended on convection, which presented a low heat transfer capacity, and the wall temperature increased rapidly with the increasing heat flow density. When the heat flow density reached the starting point (105 °C), distinct boiling began to appear on C1-3; thus, the starting point is called the initial boiling point of C1-3.

(2) Two-phase boiling stage (boiling zone)

In the two-phase boiling zone of C1-3, many bubbles began to appear on the sample surface, intense heat exchange occurred, and the increasing speed of wall temperature considerably decreased (Figure 13). For the smooth plate, wall temperature increased rapidly because the nonuniform lattice structure could effectively separate the bubbles escaping from the pores and prevent bubble coalescence due to the increasing or decreasing distribution of pores and the large spacing. The pores exhibited the capillary effect, which was conducive to liquid replenishment and delayed the critical heat flux (CHF). Therefore, C1-3 with a large surface area and rough surface exhibited dense nucleation sites, a high bubble escape frequency, and an enhanced heat exchange efficiency. When the heat flow density reached 110 W/cm^2^, the wall temperature of C1-3 rapidly increased. This heat flow density was CHF, which reached 244% of CHF for the smooth plate.

### 5.2. Effect of Cell Type on Boiling Heat Transfer

**Figure 14:**
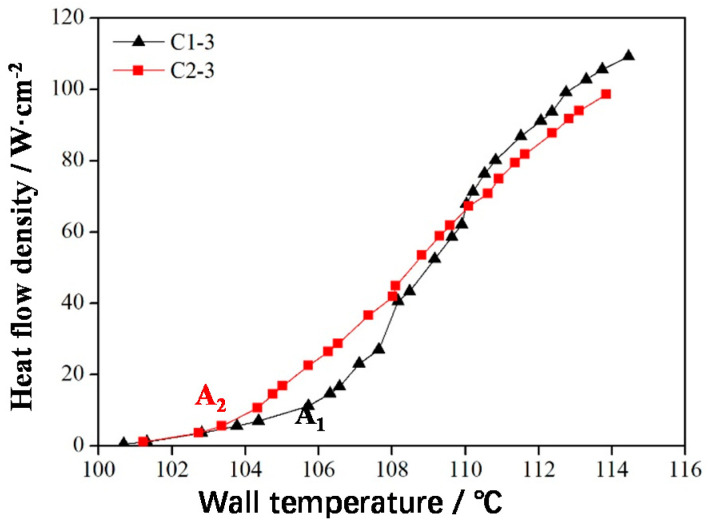
Saturated boiling curves of frame-type and FCC-type lattice structures.

The saturated boiling curves of the frame-type C1-3 and FCC-type C2-3 samples are shown in Figure 14. In the boiling heat transfer experiment, the sample placement direction was consistent with the SLM direction. The initial boiling temperature of C1-3 (approximately 105 °C, the initial boiling point was A_1_) was higher than that of C2-3 (approximately 104 °C, the initial boiling point was A_2_) because when the pore distributions of C1-3 and C2-3 were consistent, the porosity of the FCC sample, C2-3, was large. Therefore, the surface area of C2-3 was large and exhibited numerous nucleation sites.

From the starting point to boiling point under the same heat flux, the wall temperature of C2-3 was lower than that of C1-3, indicating that the heat transfer performance of C2-3 was better than that of C1-3 (Figure 14). This result was obtained because at this stage, few bubbles existed and were independent; large bubbles did not form. At this stage, the surface area was the primary factor affecting heat transfer. FCC sample C2-3 had a large surface area and exhibited numerous nucleation sites, thus strongly enhancing boiling heat transfer. After the boiling point, frame sample C1-3 demonstrated relatively better heat transfer performance when the bubble escape resistance became the main factor affecting heat transfer because many coalescing bubbles were present at this stage. The bubble escape channels of C1-3 were continuous and ran through the whole lattice structure; hence, the bubble escape resistance was small. The CHF (110 W/cm^2^) of C1-3 was 10% higher than that of C2-3 (100 W/cm^2^).

### 5.3. Effect of Nonuniform Frame-Type and FCC-Type Lattice Structures on Boiling Heat Transfer

**Figure 15:**
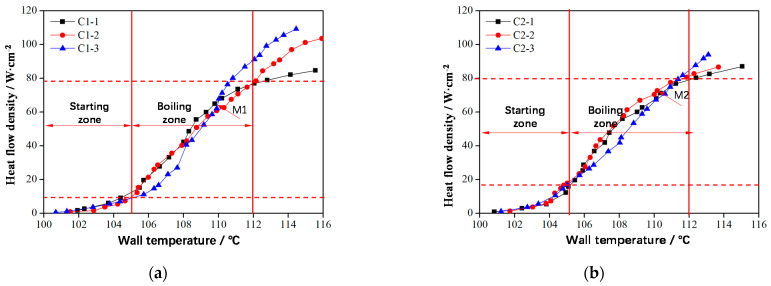
Saturated boiling curves of lattice structures. (**a**) Frame-type and (**b**) FCC-type.

The smaller the pore size of the nonuniform lattice structure, the larger the contact area between bubbles and columns, leading to a higher bubble escape resistance. However, a smaller pore size corresponds to a greater capillary force during boiling, which is conducive to liquid replenishment and delays CHF arrival. Therefore, the nonuniform lattice structure design with small and large holes in the lower and upper layers, respectively, is advantageous. The small holes in the lower layer can increase the capillary force. Moreover, the bubbles at the lower layer only separate from the column and exhibit a small volume; thus, the resistance in the small pores is limited. When the bubbles rise to the upper layer and grow in volume, the large pores can reduce the resistance [28]. The saturated boiling curve of this nonuniform lattice structure is shown in Figure 15. In the single-phase convection stage, the boiling curves of C1 frame types exhibited small variations, and the initial boiling points were almost equal because their surface areas were the same (Figure 15a).

**Figure 16:**
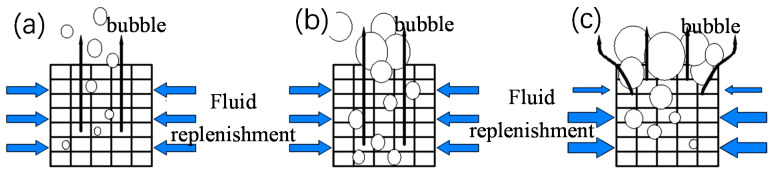
Boiling of the nonuniform frame lattice structure. (**a**) Stage I: The bubbles are independent of each other, (**b**) Stage II: The bubbles begin to accumulate, and (**c**) Stage III: The bubbles cover the upper layer of the sample.

In the two-phase boiling stage (Figure 15a), before Point M_1_, the heat flow density of the nonuniform frame structure C1-2 was low, and the bubbles were independent of each other (Figure 16a). Afterward, additional bubbles appeared and began to merge (Figure 16b); thus, the heat flow density of nonuniform lattice structures C1-1 and C1-2 in the boiling initiation area was higher than that of the uniform frame structure C1-3. After Point M_1_, with the increased in the heat flux, bubbles accumulated until the upper layer of the sample was covered (Figure 16c); hence, the upper layer liquid could not be replenished, which deteriorated heat exchange conditions. At this stage, heat transfer mostly depended on the lower layer. Because the upper layer of the uniform lattice structure had a large bubble escape channel and suitable heat transfer conditions, its overall heat transfer effect was better than that of nonuniform structures. However, the CHF of C1-2 (approximately 105 W∙cm^−2^) was considerably larger than that of C1-1 (approximately 85 W∙cm^−2^) because the lower layer of C1-2 had many small pores (Figure 16a,c), which increased the capillary force and facilitated liquid replenishment, thus delaying CHF.

**Figure 17:**
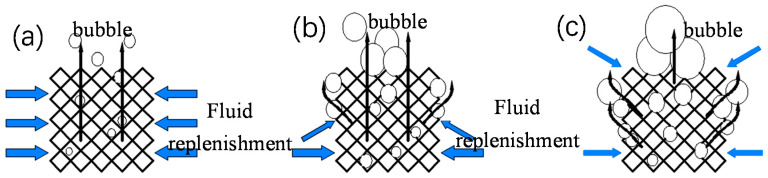
Boiling of the nonuniform FCC lattice structure. (**a**) Stage I: The bubbles are independent of each other, (**b**) Stage II: The bubbles begin to accumulate, and (**c**) Stage III: The bubbles cover the upper layer of the sample.

**Figure 18:**
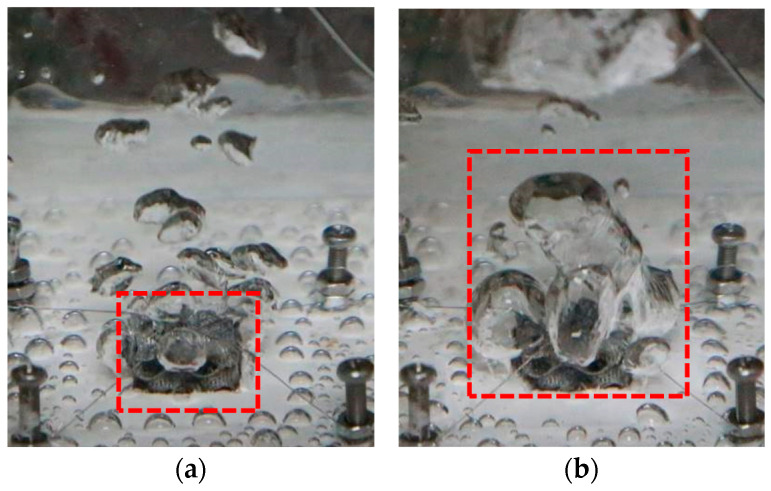
Bubble diagrams of the two-phase boiling heat transfer at Point M. (**a**) Nonuniform frame lattice structure and (**b**) Nonuniform FCC lattice structure.

The trend for the heat transfer conditions of the nonuniform FCC lattice sample was consistent with that of the uniform FCC lattice structure. However, the nonuniform FCC lattice M_2_ point (approximately 75 W∙cm^−2^) appeared slightly later than the uniform FCC lattice M_1_ point (approximately 65 W∙cm^−2^) of the frame lattice structure because the bubble escape channels of the frame-type lattice structure were straight and large, which facilitated the bubbles to escape from the top, coalesce, and cover the upper sample (Figure 16). Therefore, the heat exchange conditions of the upper layer were relatively better. The channels of the FCC lattice structure were inclined; thus, the bubbles tended to escape from the side (Figure 17b), thereby delaying the deterioration of the upper-layer heat transfer conditions. Therefore, the heat transfer effect of the nonuniform FCC lattice structure was better than that of the frame lattice structure. At the optimal boiling heat transfer temperature of 108 °C, the boiling heat flux of the FCC lattice structure (approximately 50 W∙cm^−2^) was 25% larger than that of the frame lattice structure (approximately 40 W∙cm^−2^). In addition, at high heat fluxes, the liquid replenishment channels of the FCC sample were highly dense (Figure 17c), which was conducive to liquid replenishment (Figure 18b).

## 6. Conclusions

In this study, uniform and nonuniform lattice structures were created through SLM for two-phase microchannel liquid-cooled heat exchangers. The calculation and preparation of nonuniform lattice structures were discussed, and the influence of SLM characteristics on the boiling heat transfer effect of the sample was investigated, leading to the following conclusions:

(1) Powder bonding was frequent on the surface of the SLM-formed nonuniform lattice samples, and the Ra of the side surface profile was 10–16 μm. Thus, many potential nucleation sites could be formed to reduce initial boiling temperature. Slagging, the step effect, and powder bonding can be observed during SLM, resulting in smaller porosities than the design porosity values.
(2) Due to the gradient increasing or decreasing the distribution and large spacing of pores in the uniform lattice structure, the bubbles escaping from the pores could be effectively separated to prevent bubble coalescence. The pores also exhibited a capillary effect, facilitating liquid replenishment and delaying CHF. The heat flux in the boiling zone was a minimum of 244% higher than that of the traditional plate structure; the critical heat flux density of the lattice structure was 110 W∙cm^−2^, and the critical heat flux density of the traditional flat plate was 45 W∙cm^−2^.
(3) The surface area of the frame lattice sample was small; therefore, its initial boiling temperature (approximately 105 °C) was higher than that of the FCC lattice sample (approximately 104 °C). The bubble escape channels of the frame lattice structure ran vertically through the whole structure, exhibiting the small escape resistance and orderly liquid replenishment; thus, its CHF was >10% for the FCC cell structure.
(4) For FCC-type cell structures, the heat transfer trends of the nonuniform and uniform samples were consistent. However, the heat transfer effect of the nonuniform FCC lattice structure was better than that of the frame-type cell structure. At the optimal boiling heat transfer temperature, the boiling heat flux of the FCC-type lattice structure was 25% higher than that of the frame-type lattice structure.

## Figures and Tables

**Table 1:** Primary working parameters of the laser.

Parameter	Setting
Rated output power/W	250
Working mode	Continuous/Modulated
Centre wavelength/nm	1080
Output power fluctuation	<3%
Minimum spot diameter/mm	0.06

**Table 2:** Chemical composition of the CuSn10 powder.

Element	Assay Value/wt.%
Sn	9.88
O	0.039
Cu	Bal.
Impurities	≤0.2

**Table 3:** Structural parameters of nonuniform lattices.

No.	Lattice Type	Column Diameter/mm	Porosity/%	Surface Area/mm^2^	Size/mm
C1-1	Frame-type	0.6–1.2	66.86	4175	15 × 15 × 15
C1-2		0.885	67.02	4331	15 × 15 × 15
C1-3		0.895	67.02	4279	15 × 15 × 15
C2-1	FCC-type	0.6–1.2	66.86	4191	15 × 15 × 15
C2-2		0.895	66.88	4306	15 × 15 × 15
C2-3		0.895	67.06	4280	15 × 15 × 15

**Table 4:** SLM process parameters.

Laser Power/W	Scanning Speed/mm·s^−1^	Scanning Spacing/mm	Layer Thickness/mm	Scanning Mode
210	1300	0.07	0.05	Line by line

**Table 5:** Sample porosity.

No.	Designed Porosity/%	Actual Porosity/%	Relative Error/%
C1-1	67	61	9.0
C1-2	67	61	8.3
C1-3	67	61	8.4
C2-1	67	62	7.7
C2-2	67	62	7.2
C2-3	67	62	7.4

## Data Availability

Not applicable.
